# Gut microbiota profile of COVID-19 patients: Prognosis and risk stratification (MicroCOVID-19 study)

**DOI:** 10.3389/fmicb.2022.1035422

**Published:** 2022-11-22

**Authors:** José Guilherme Nobre, Mariana Delgadinho, Carina Silva, Joana Mendes, Vanessa Mateus, Edna Ribeiro, Diogo Alpuim Costa, Miguel Lopes, Ana Isabel Pedroso, Frederico Trigueiros, Maria Inês Rodrigues, Cristina Lino de Sousa, Miguel Brito

**Affiliations:** ^1^Faculty of Medicine, Lisbon University, Lisbon, Portugal; ^2^Faculdade de Medicina, Instituto de Saúde Ambiental, Universidade de Lisboa, Lisboa, Portugal; ^3^PTSurg – Portuguese Surgical Research Collaborative, Lisbon, Portugal; ^4^H&TRC- Health and Technology Research Center, ESTeSL- Escola Superior de Tecnologia da Saúde de Lisboa, Instituto Politécnico de Lisboa, Lisbon, Portugal; ^5^Centro de Estatística e Aplicações, Universidade de Lisboa, Lisbon, Portugal; ^6^Breast Cancer Unit, CUF Oncologia, Lisbon, Portugal; ^7^Faculdade de Ciências Médicas, NOVA Medical School, Lisbon, Portugal; ^8^Departamento de Pneumologia, Hospital Garcia de Orta, Almada, Portugal; ^9^Serviço de Medicina Intensiva, Hospital de Cascais Dr. José de Almeida, Cascais, Portugal; ^10^Departamento de Medicina Interna I, Centro Hospitalar Lisboa Norte – Hospital de Santa Maria, Lisbon, Portugal; ^11^Departamento de Medicina Interna, Hospital da Luz, Lisbon, Portugal

**Keywords:** COVID-19, microbiota, microbiome, dysbiosis, risk stratification, prognosis, next generation sequencing

## Abstract

**Background:**

Gut microbiota is intrinsically associated with the immune system and can promote or suppress infectious diseases, especially viral infections. This study aims to characterize and compare the microbiota profile of infected patients with SARS-CoV-2 (milder or severe symptoms), non-infected people, and recovered patients. This is a national, transversal, observational, multicenter, and case–control study that analyzed the microbiota of COVID-19 patients with mild or severe symptoms at home, at the hospital, or in the intensive care unit, patients already recovered, and healthy volunteers cohabiting with COVID-19 patients. DNA was isolated from stool samples and sequenced in a NGS platform. A demographic questionnaire was also applied. Statistical analysis was performed in SPSS.

**Results:**

Firmicutes/Bacteroidetes ratios were found to be significantly lower in infected patients (1.61 and 2.57) compared to healthy volunteers (3.23) and recovered patients (3.89). Furthermore, the microbiota composition differed significantly between healthy volunteers, mild and severe COVID-19 patients, and recovered patients. Furthermore*, Escherichia coli*, *Actinomyces naeslundii*, and *Dorea longicatena* were shown to be more frequent in severe cases. The most common COVID-19 symptoms were linked to certain microbiome groups.

**Conclusion:**

We can conclude that microbiota composition is significantly affected by SARS-CoV-2 infection and may be used to predict COVID-19 clinical evolution. Therefore, it will be possible to better allocate healthcare resources and better tackle future pandemics.

## Introduction

The coronavirus disease 2019 (COVID-19) was declared a pandemic by WHO on 11^th^ March 2020 (World Health Organization (WHO), 2022), and is caused by the severe acute respiratory syndrome coronavirus 2 (SARS-CoV-2) transmitted to the respiratory tract through droplets, respiratory secretions, and direct contact. It was also reported that COVID-19 could be found in the fecal material of patients (Guo et al., 2020) COVID-19 links to cells through the angiotensin-converting-enzyme 2 (ACE2) protein, which is present in lung alveolar epithelial cells and enterocytes of the small intestine. The main clinical symptoms are dry cough, fever, fatigue, sputum production, shortness of breath, sore throat, and headache. Also, some patients present diarrhea and vomiting (Guo et al., 2020).

Gut microbiota is intrinsically involved in the development and appropriate function of the immune system. A eubiotic microbiota is crucial for facing infectious diseases more efficiently, with less aggressiveness, and a better prognosis (Van Den Elsen et al., 2017). One of the main mechanisms promoted by gut microbiota is limiting access to the intestinal epithelium. This happens through immunoglobulin A production, which binds to microbes at mucosal surfaces, neutralizes toxins, and contributes to microbial tolerance (Van Den Elsen et al., 2017). It was shown that the gut microbiota is also vital in establishing immune defenses in other microbial sites, such as the lungs. Reactive oxygen species (ROS)-mediated defense of alveolar macrophages in *Klebsiella pneumoniae* infection is modulated by gut microbiota (Van Den Elsen et al., 2017).

Regarding respiratory infections, a study on pigs has demonstrated that gut microbiota can have a massive impact on respiratory pathogens. Firstly, it was observed that the microbiota profile was associated with clinical outcomes after infection with Porcine Reproductive and Respiratory Syndrome Virus (PRRSV) and Porcine Circovirus Type 2 (PCV2). Pigs with increased microbiota diversity showed reduced clinical signs and symptoms and disease severity (Niederwerder et al., 2016). Secondly, it was assessed that microbiota characteristics present at the time of virus exposure were related to outcome after co-infection with PRRSV and PCV2. It was observed that pigs with higher microbiota diversity, reduced *Methanobacteriaceae* species, and enhanced *Ruminococcaceae* and *Streptococcaceae* species demonstrated reduced viral replication and less severe pneumonia (Ober et al., 2017). Moreover, microbiota transplantation was evaluated as a prophylactic measure to reduce co-infection by PRRSV and PCV2. Furthermore, it was observed that transplanted pigs had lower morbidity and mortality rates, combined with enhanced antibody production (Niederwerder et al., 2018). In addition, other respiratory viruses, like the influenza virus, are directly or indirectly suppressed by microbiota. Thus, evaluating the microbiota profile is crucial to better understand the clinical outcomes of patients, but interventions that modulate the microbiota also appear to be essential to reduce morbidity and mortality, decrease disease severity and even increase the antibody response to infection (Libertucci and Young, 2019; Li et al., 2019).

Recently, several microbiota studies were performed on COVID-19 patients. As it has been widely studied, SARS-CoV-2 has a high affinity for ACE2 receptors since it is the primary entry route for cell entrance. These ACE2 receptors are expressed in numerous types of cells throughout the organism, such as epithelial cells in the lungs, small intestine, colon, among others. SARS-CoV-2 virus, intending to increase its replication efficiency, downregulates ACE2 receptors (Lee et al., 2020; Villapol, 2020). Hence, either the positive or negative actions are suppressed, leading to innumerous deleterious consequences, like the overexpression of the RAS-dependent pathway, exacerbating cardiovascular risk, and promotion of microbiota dysbiosis, enhancing the possibility of a cytokine storm as a complication of COVID-19 (Viana et al., 2020). This imbalance of RAS/ACE2 leads to a poorer prognosis (Dijkman et al., 2012; Chen et al., 2020; Villapol, 2020).

As described previously, COVID-19 patients tend to have microbiota dysbiosis (Venzon et al., 2022). A few studies have demonstrated that some bacterial species are correlated with disease severity, which can hold the promise of using them as predictive biomarkers. However, plenty of questions regarding microbiota and COVID-19 remain unanswered. Hence, in an attempt to help demystify this interplay, we aimed with this study to evaluate, correlate and compare gut microbiota profiles of in and outpatients infected with SARS-CoV-2, recovered patients from COVID-19 infection, and a control group of uninfected volunteers in order to determine a potential gut microbiota profile signature of these individuals.

## Materials and methods

### Study design and participants

A prospective observational study was conducted between June 2020 and June 2021, in different centers in Lisbon (Portugal) to compare the gut microbiota profile of outpatients and inpatients infected with COVID-19, recovered patients, and healthy controls. The study protocol was performed in accordance with the declaration of Helsinki guidelines and with ethics approval from the institutional review boards of each involved institution (ESTeSL - CE-ESTeSL-N°0.28–2020, HCDJA EO 14/2020, HGO, 18/06/2020, HLuz 16/07/2020,). Written informed consent from the participants and social-demographic questionnaires were obtained before sample collection. Only patients who met the following inclusion criteria were eligible for this study: (1) age equal to or greater than 18 years old; (2) for the infected group, a COVID-19 diagnosis confirmed in a central laboratory in the last 48 h; (3) for the uninfected group, cohabiting with an infected patient recovering at home, with a negative test in the last 48 h; (4) for the recovered group, having a negative test in the last 48 h, after cessation of symptoms, and previous confirmation of COVID-19 infection. The exclusion criteria were: (1) pregnant or lactating patients; (2) unusual clinical conditions that the responsible physician or investigator could consider influencing the gut microbiota analysis, such as chronic diseases (e.g., diabetes, cardiovascular inflammatory bowel disease). In addition, the responsible physician classified the disease severity of the infected group as mild or severe, based on WHO Clinical Progression Scale. None of the individuals had previously been immunized against SARSCOV2. Infected participants were patients receiving care at the Hospital Santa Maria (HSM), Hospital Garcia de Orta (HGO), Hospital de Cascais Dr. José de Almeida, and Hospital das Forças Armadas (HFAR); and the samples from the recovered and control groups were from participants of ESTeSL or the nursing home “Os Amigos de Sempre.” Written informed consent from the participants and social-demographic questionnaires were obtained before sample collection.

### DNA extraction and 16S sequencing

In order to ensure sample stability during transportation and storage, the stool samples were collected using the DNA/RNA Shield Fecal Collection tubes (Zymo Research). According to the manufacturer’s instructions, microbial DNA was extracted using ZymoBIOMICS™ DNA Miniprep Kit (Zymo Research) and FastPrep-24™ homogenizer (MP Biomedicals). The NanoDrop One spectrophotometer (ThermoScientific) was used to quantify DNA samples, which were stored at −20°C.

Preparation of libraries for sequencing was performed following the 16S Metagenomic Sequencing Library Preparation Illumina document. In this step, we used the KAPA HiFi HotStart Ready Mix (Roche) and the following primers to amplify V3-V4 hypervariable regions of the bacterial 16S rRNA gene: 16F (5′- TCGTCGGC AGCGTCAGAT GTGTATAAGAG ACAGCCTA CGGGNGGC WGCAG −3′) and 16R (5′- GTCTCGTGGGC TCGGAGATGTGTA TAAGAGAC AGGACTACHVG GGTATC TAATCC -3′) (Klindworth et al., 2013). Following PCR amplification, the dsDNA HS assay kit for the Qubit 3.0 fluorometer (Thermo Fisher Scientific) and the High Sensitivity D1000 ScreenTape and Reagents for TapeStation 4200 (Agilent Technologies) were used to determine DNA concentration and amplicon lengths, respectively.

Illumina index adapters were added for each sample, and all purification steps were carried out by using AMPure XP magnetic beads (Beckman Coulter). The resulting indexed libraries were checked on TapeStation and quantified on Qubit. Then, an equimolar pool of 4 nM was prepared for further denaturalization, dilution to 2 pM, and sequencing on the NextSeq550 instrument (Illumina) with 2 × 151 bp paired-end reads. After this procedure, the software generated FASTQ files.

### Quality control, taxonomic assignment, and statistical analysis

Microbiota taxonomic profiles were generated using the EzBioCloud MTP pipeline and EzBioCloud 16S database PKSSU4.0 (Yoon et al., 2017). The single-end reads were uploaded in this software for data quality checking, trimming primers and filtering out sequences of low quality. The UCHIME algorithm was applied to check and remove chimeras. All sequences that did not match any reference with at least 97% similarity cutoff were clustered using UCLUST method with a 97% cutoff.

Using OTU information, several alpha and beta diversity indices were calculated. These include species richness estimators (such as CHAO1, ACE, and Jackknife) and diversity indices (Simpson and Shannon). The alpha-diversity statistical differences were calculated with the Kruskal–Wallis test using SPSS version 27 (IBM). Significant differences between the group patient’s microbiota at various taxonomic levels were assessed using the Statistical Analysis of Metagenomic Profiles (STAMP) software package v2.1.3 (Parks et al., 2014) and applying the Welch’s test. *p* < 0.05 were considered statistically significant.

## Results

### Demographical and clinical characterization

In the present study, a total of 87 questionnaires were collected in a cohort of healthy controls (*n* = 7), COVID-19 infected patients (*n* = 35) (either isolated at home or hospitalized) and recovered patients (*n* = 45) between June 2020 and June 2021. All the demographic and clinical data of these patients are organized in Table 1. A significant difference in age and race parameters was observed in our sampled population. We also noticed that the recovered and control group subjects were more physically active than those in the infected groups, however they were not age-matched.In four of the clinical parameters, the severe group answered yes more frequently, which were: the occurrence of bowel movements modification (40.7%), presence of chronic diseases (88.9%), chronic medication intake (81.5%), and antibiotic usage in the last 6 months (51.9%). The most common symptoms in the severe group were fever (74.1%), dyspnea (44.4%), and dry cough (37%) in this order, whereas the mild group suffered more from the dry cough symptom (62.5%).

**Table 1:** Demographic and clinical characteristics of the study population. Statistical analysis was performed with ANOVA/Kruskal–Wallis test or Chi-square/Fischer test.

	Control group (*n* = 7)	Mild COVID-19 (*n* = 8)	Severe COVID-19 (*n* = 27)	Recovered (*n* = 45)	*p*-value
Age (years)	33.4 ± 13.2	64.5 ± 22.5	63.6 ± 16.5	56.3 ± 26.8	0.001^1^
Gender (% female)	57.1% (4)	37.5% (3)	33.3% (9)	77.8% (25)	
*Race (%)*					
European	71.4% (5)	87.5% (7)	77.8% (21)	80% (36)	0.002^2^
African		12.5% (1)	14.8% (4)		
Gipsy			7.4% (2)	2.2% (1)	
*Diet (%)*					
Mediterranean	71.4% (5)	87.5% (7)	74.1% (20)	88.9% (40)	
Fast-food		12.5% (1)	3.7% (1)	2.2% (1)	
Vegetarian/vegan	14.3% (1)			4.4% (2)	
Physical exercise (% yes)	28.6% (2)	0% (0)	11.1% (3)	24.4% (11)	<0.001^2^
Alcohol consumption (% yes)	14.3% (1)	12.5% (1)	11.1% (3)	6.7% (3)	0.335^2^
Smoking habits (% yes)	0% (0)	0% (0)	3.7% (1)	4.4% (2)	0.123^2^
Bowel movements modification (last 6 months) (% yes)	14.3% (1)	25% (2)	40.7% (11)	35.6% (16)	<0.001^2^
Chronic diseases (% yes)	28.6% (2)	50% (4)	88.9% (24)	46.7% (21)	<0.001^2^
Chronic medication (% yes)	28.6% (2)	37.5% (3)	81.5% (22)	55.6% (25)	<0.001^2^
Antibiotic usage (% yes)	14.3% (1)	25% (2)	51.9% (14)	4.4% (2)	<0.001^2^
Probiotic usage (% yes)	28.6% (2)	12.5% (1)	11.1% (3)	4.4% (2)	<0.001^2^
BCG vaccination (% yes)	100% (7)	75% (6)	29.6% (8)	73.3% (33)	<0.001^2^
PNV vaccination (% yes)	100% (7)	100% (8)	51.9% (14)	82.2% (37)	<0.001^2^
Anti-pneumococcal vaccination (% yes)	100% (7)	12.5% (1)	7.4% (2)	0% (0)	<0.001^2^
Flu vaccination (% yes)	28.6% (2)	12.5% (1)	22.2% (6)	46.7% (21)	0.094^2^
*Symptoms*					
None	100% (7)	12.5% (1)	3.7% (1)		
Dry cough		62.5% (5)	37% (10)	13.3% (6)	
Dyspnea		25% (2)	44.4% (12)	2.2% (1)	
Fever		37.5% (3)	74.1% (20)	6.7% (3)	
(%)Sputum		12.5% (1)	11.1% (3)	2.2% (1)	
Headache		25% (2)	14.8% (4)	4.4% (2)	
Malaise		87.5% (6)	29.6% (8)	4.4% (2)	
Muscular/articular pain		37.5% (3)	29.6% (8)	11.1% (5)	
Loss of smell/taste		62.5% (5)	7.4% (2)	8.9% (4)	
Throat pain		12.5% (1)	7.4% (2)	8.9% (4)	
Family aggregate infection (% yes)	57.1% (4)	37.5% (3)	37% (10)	28.9% (13)	
*Ventilatory support (%)*					
High throughput flow	0% (0)	0% (0)	11.1% (3)	0% (0)	
Mechanical ventilation			18.5% (5)		
Without ventilation			59.3% (16)		
Sequels/complications (% yes)	0% (0)	0% (0)	7.4% (2)	11.1% (5)	

1 ANOVA/Kruskal–Wallis test.

2 Chi-square/Fischer test.

All the bold values in the value of p column represent statistically significant results with a value of *p* < 0.05.

### Association between fecal microbiota profile and COVID-19 severity

**Figure 1:**
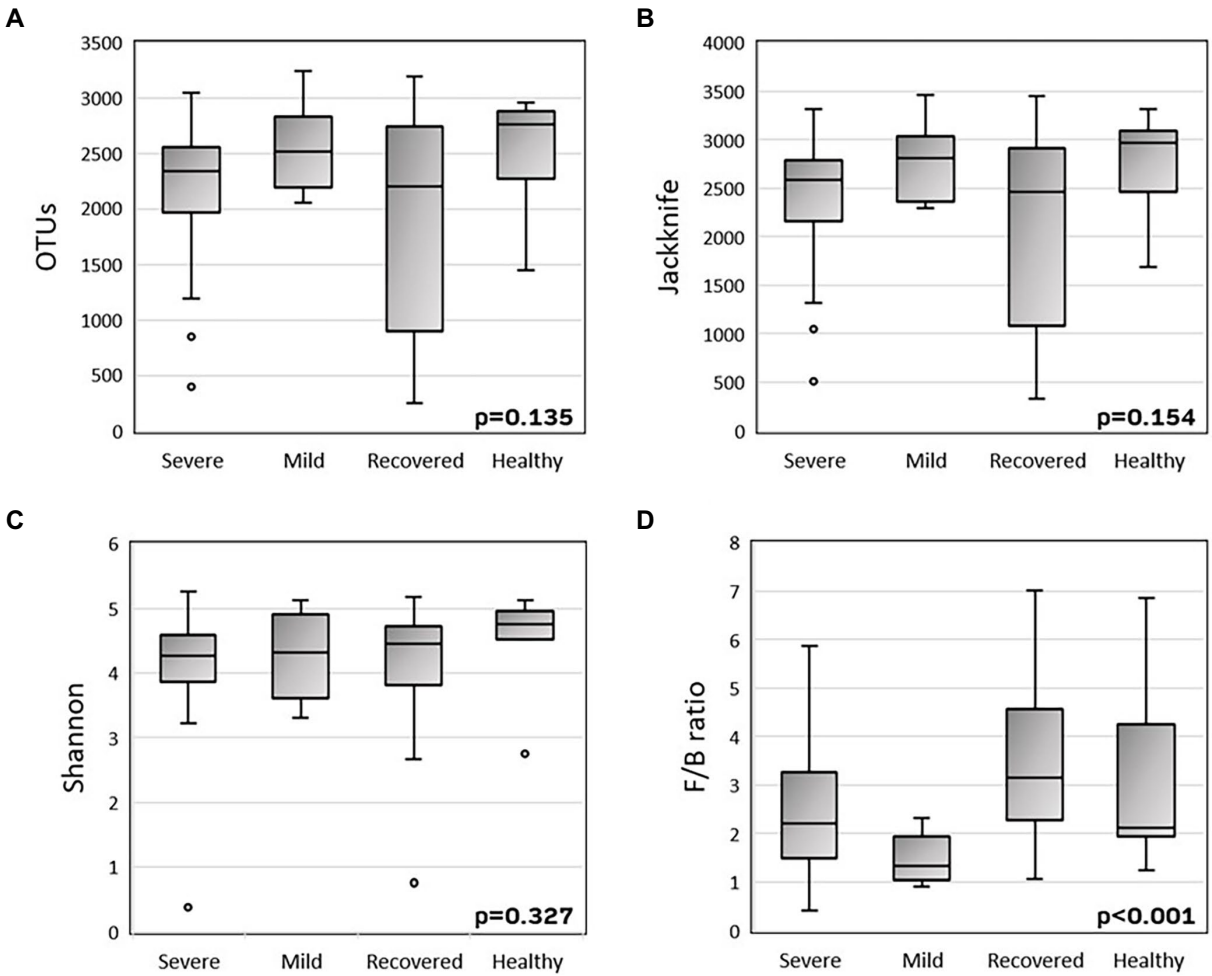
Boxplots of alpha-diversity **(A-C)** and Firmicutes/Bacteroidetes ratio **(D)**. Alpha-diversity was measured by the number of observed OTUs (A), Jackknife index **(B)** and Shannon index **(C)**. P-values were calculated with the Kruskal-Wallis test using SPSS v27.

To explore the alpha-diversity within the different groups (severe, mild, recovered, and healthy), we measured the species richness calculated by the Jackknife index and the number of observed OTUs, and the diversity calculated by the Shannon index (Figure 1). There were no statistically significant variations in the variability of these indices among the four groups. The Firmicutes/Bacteroidetes ratio was significantly different (*p* < 0.001) between the four groups, with a decreased mean ratio in the COVID-19 patients, with both mild (1.61) and severe (2.57) symptoms, when compared with the recovered (3.89) and healthy (3.23) volunteers.

**Figure 2:**
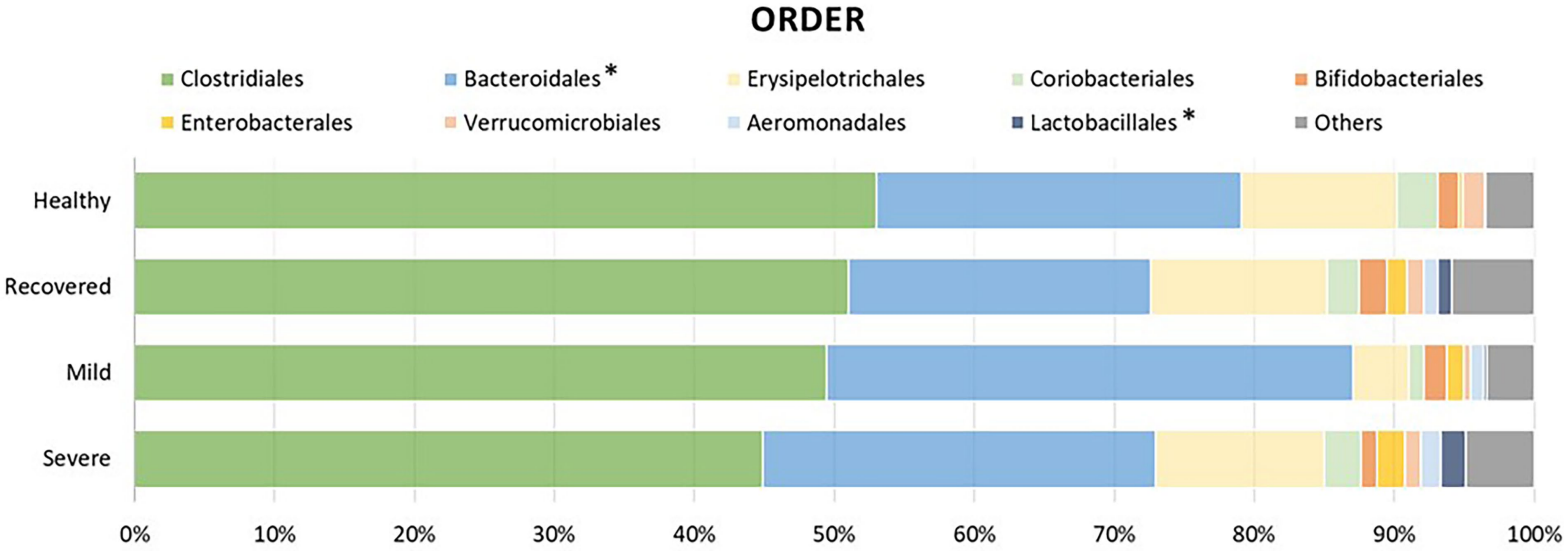
Relative abundance of the most prevalent bacterial orders between the different groups of this study: healthy volunteers, recovered patients, and COVID-19 infected patients with mild or severe symptoms. The asterisk symbol represents the statistically significant taxonomic orders.

**Figure 3:**
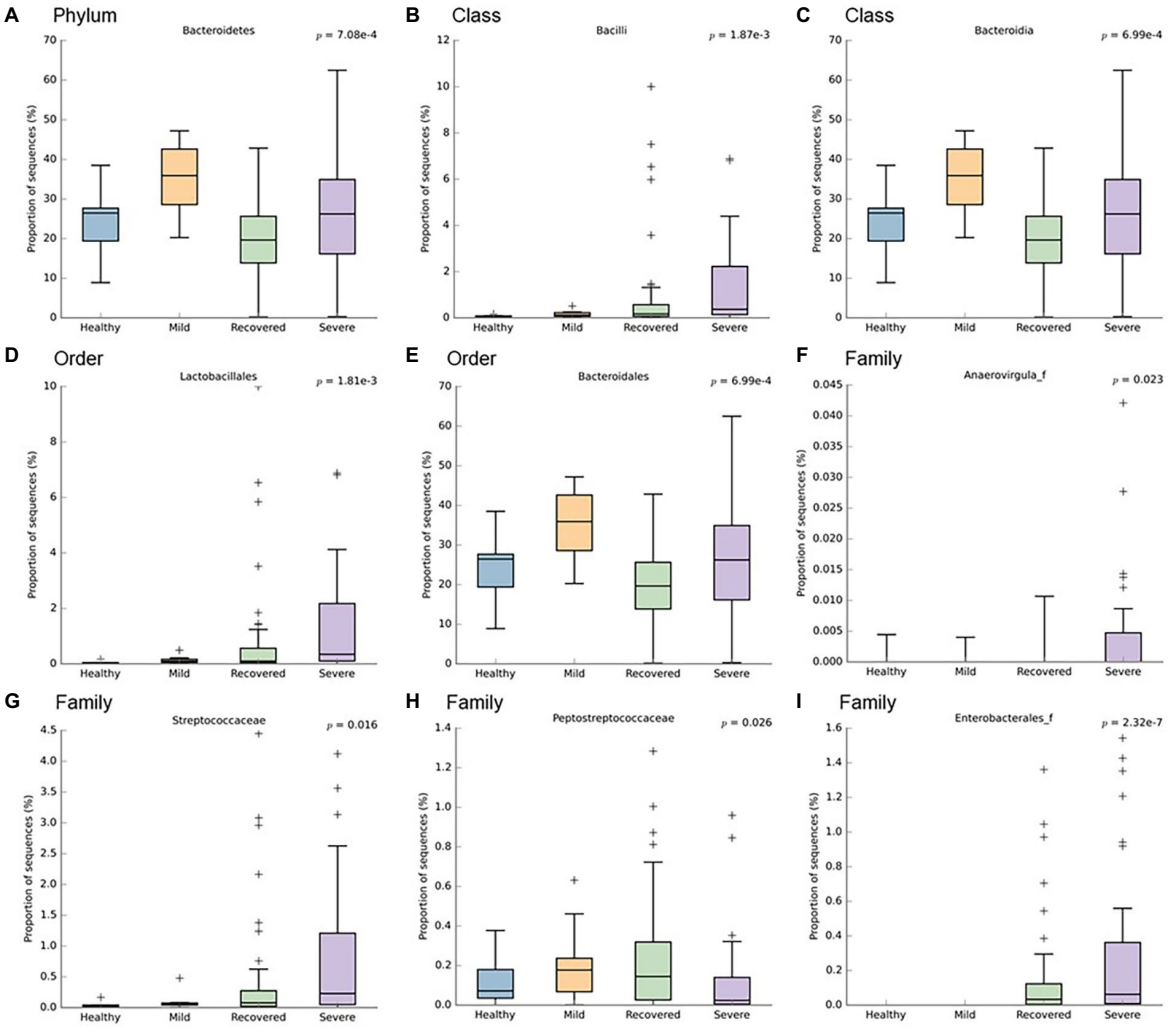
Boxplots representing the relative abundance of significant bacteria: **(A)** *Bacteroidetes*, **(B)** *Bacilli*, **(C)** *Bacteroidia*, **(D)** *Lactobacillales*, **(E)** *Bacteroidales*, **(F)** *Anaerovirgula*, **(G)** *Streptococcaceae*, **(H)** *Peptostreptococcaceae*, **(I)** *Enterobacterales*. This analysis was performed with Welch’s t-test and an effect size filter of 0.05, to compare the abundance differences between the study’s groups.

In order to identify which bacteria responded to the influence of disease severity, their relative abundance was analyzed (Figures 2, 3). It is possible to observe a higher abundance of *Bacteroidetes phylum* (*p* < 0.001) and *Bacteroidia* class (*p* < 0.001) in the mild and severe groups. Also at the class level, the proportion of sequences of *Bacilli* was significantly lower in the mild and healthy volunteers (*p* < 0.001). At the order level, the severe and recovered groups had a higher abundance of *Lactobacillales* (*p* < 0.001) and the mild group had more *Bacteroidales* (*p* < 0.001). There were three families with higher values in the severe group: *Anaerovirgula* (*p* = 0.023)*, Streptococcaceae* (*p* = 0.016), and *Enterobacterales* (*p* < 0.001); however, the recovered group had more *Peptostreptococcaceae* (*p* = 0.026).

**Figure 4:**
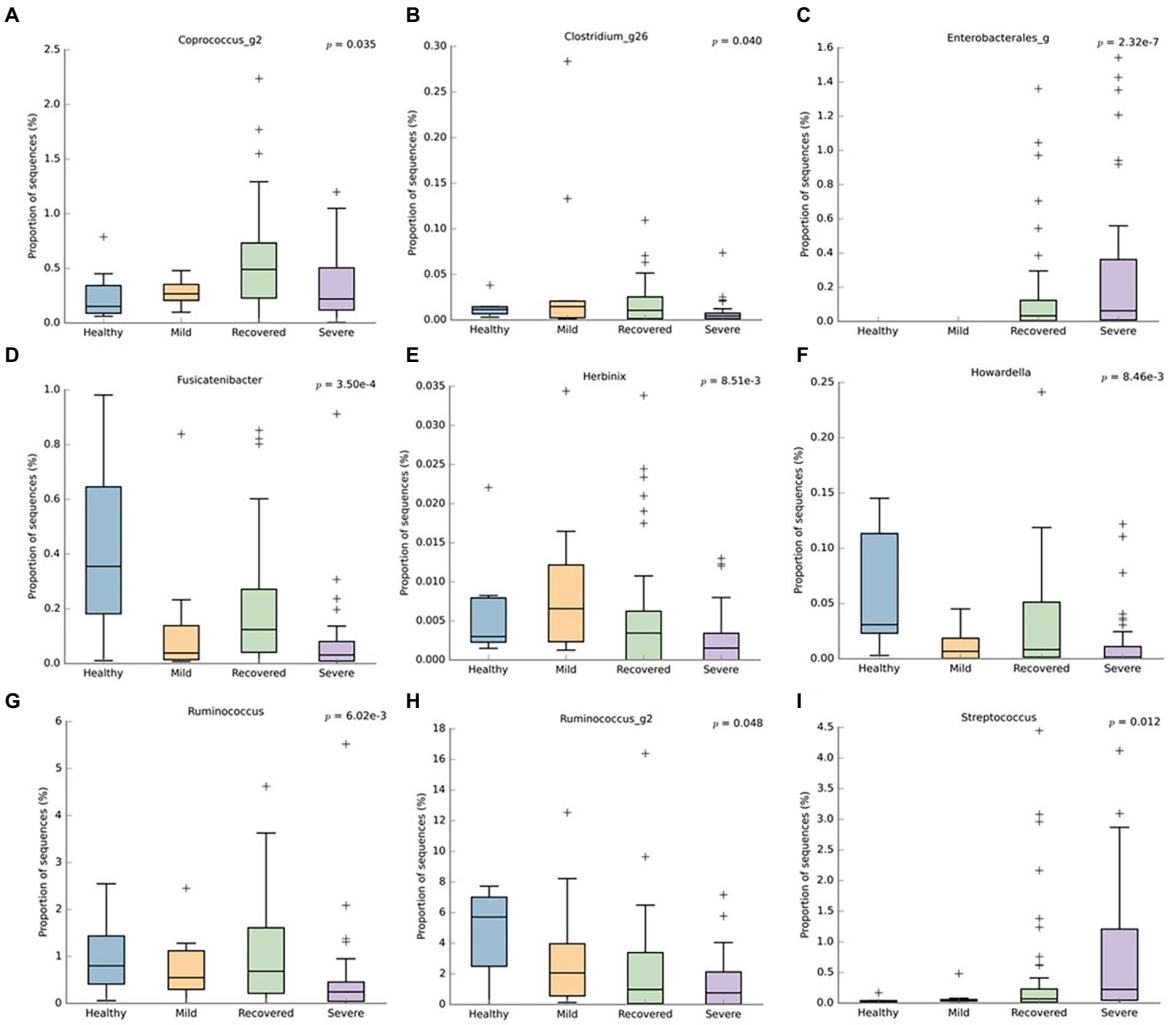
Boxplots representing the relative abundance of significant bacterial genera: **(A)** *Coprococcus_g2*, **(B)** *Clostridium_g26*, **(C)** *Enterobacterales*, **(D)** *Fusicatenibacter*, **(E)** Herbinix, **(F)** *Howardella*, **(G)** *Ruminococcus*, **(H)** *Ruminococcus_g2*, **(I)** *Streptococcus*. This analysis was performed with Welch’s t-test and an effect size filter of 0.05, to compare the abundance differences between the groups.

Regarding the genus level (Figure 4), the recovered and healthy groups had a higher abundance of *Fusicatenibacter* (*p* < 0.001)*, Howardella* (*p* < 0.001), and *Ruminococcus* (*p* < 0.001). Conversely, the severe group had a superior abundance of *Enterobacterales* (*p* < 0.001) and *Streptococcus* (*p* = 0.012), followed by the recovered group. Besides the lack of these last-mentioned bacteria in the healthy group, the *Coprococcus_g2* (*p* = 0.035) and *Clostridium_g26* (*p* = 0.040) were also lower in this particular group. Additionally, the severe group had a lower abundance of *Herbinix* (*p* < 0.001)*, Ruminococcus* (*p* < 0.001), and *Ruminoccus_g2* (*p* = 0.048).

**Figure 5:**
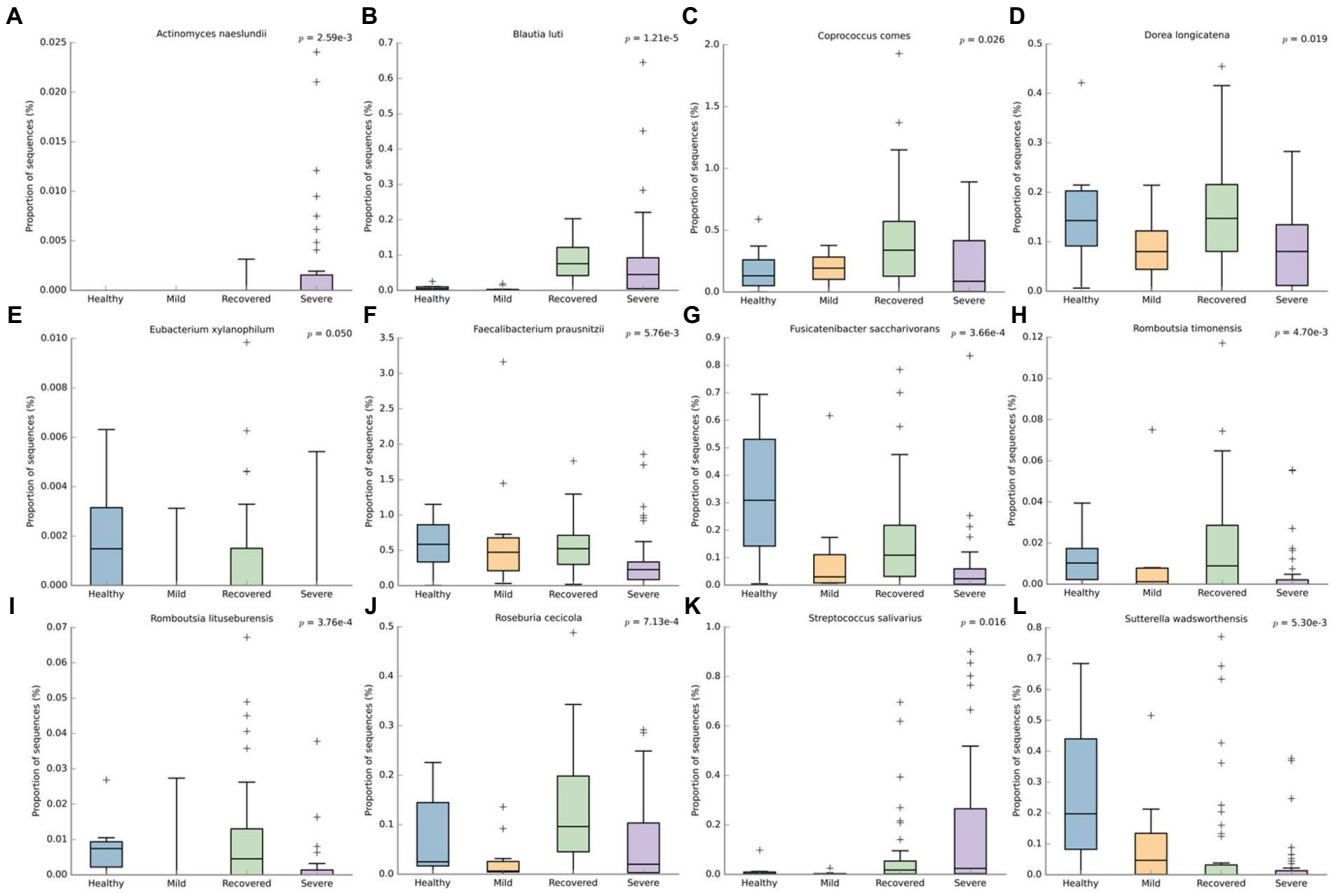
Boxplots representing the relative abundance of significant bacterial species: **(A)** *Actinomyces naeslundii*, **(B)** *Blautia luti*, **(C)** *Coprococcus comes*, **(D)** *Dorea longicatena*, **(E)** *Eubacterium xylanophilum*, **(F)** *Faecabacterium prausnitzii*, **(G)** *Fusicatenibacter saccharivorans*, **(H)** *Romboutsia timonensis*, **(I)** *Romboutsia lituseburensis*, **(J)** *Roseburia cecicola*, **(K)** *Streptococcus salivarius*, **(L)** *Sutterella wadsworthensis*. This analysis was performed with We’ch’s t-test and an effect size filter of 0.05, to compare the abundance differences between the groups.

At the species level (Figure 5), *Actinomyces naeslundii* (*p* < 0.001) and *Streptococcus salivarius* (*p* = 0.016) had a higher abudance in the severe group. The non-infected individuals (healthy and recovered) had more reads for several bacteria: *Dorea longicatena* (*p* = 0.019)*, Eubacterium xylanophilum* (*p* = 0.050), *Faecabacterium prausnitzii* (*p* < 0.001), *Fusicatenibacter saccharivorans* (*p* < 0.001), *Romboutsia timonensis* (*p* < 0.001), *Romboutsia lituseburensis* (*p* < 0.001) and *Roseburia cecicola* (*p* < 0.001). Besides this, *Coprococcus comes* (*p* = 0.026) and *Blautia luti* (*p* < 0.001) had a higher abundance in the recovered and *Sutterella wadsworthensis* (*p* < 0.001) in the healthy volunteers.

Lastly, we noticed several OTUs that were only detected in the healthy and mild group, such as: *Agathobacter rectalis* (*p* < 0.001), *Alistipes senegalensis* (*p* < 0.001), *Bacteroides finegoldii* (*p* < 0.001), *B. xylanisolvens* (*p* < 0.001), *Bifidobacterium adolescentis* (*p* < 0.001), *Escherichia coli* (*p* < 0.001), *Gemmiger formicillis* (*p* < 0.001), *Pantoea agglomerans* (*p* < 0.001) and *Streptococcus parasanguinis* (*p* < 0.001). There were also two bacteria only present in the healthy volunteers: *Pseudobutyrivibrio xylanivorans* (*p* < 0.001) and *S. constellatus* (*p* < 0.001); and only one bacterium with reads in the mild group: *Rodentibacter trehalosifermentans* (*p* < 0.001).

### Do demographic characteristics influence microbiota composition and COVID-19 clinical outcomes?

Furthermore, with the aim to validate if microbiota composition modifications were exclusively due to SARS-CoV-2 infection, demographic characteristics were evaluated through univariate general linear models. Therefore, it was demonstrated that *Coprococcus* spp. was influenced by ethnicity (*p* = 0.042), physical exercise (*p* = 0.038) and sleep duration (*p* = 0.003), while *Clostridium_g26* spp.is affected by prebiotic/probiotic intake (*p* = 0.001) and intestinal habits modification (*p* = 0.047). *Enterobacterales* spp. is modified by the presence of probiotic (*p* = 0.004) as well as chronic diseases (*p* = 0.004). *Herbinix* spp. concentration might be changed by gender (*p* = 0.042), weight (*p* = 0.005) and alcohol abuse (*p* = 0.037). *Ruminococcus* are modified by long-term medications (*p* = 0.019). In terms of species, *Streptococcus salivarus* are altered in chronic diseases states (*p* = 0.044), as well as *Faecalibacterium prauznitzii* (*p* = 0.039) and *Acinectobacter naeslaundii* (*p* = 0.007). The compliance with the Portuguese national vaccination plan solely affects the concentrations of *Coprococcus* spp. (*p* = 0.038), *Clostridium_g26* spp. (*p* = 0.005) and *Eubacterium xylanophilum* (*p* = 0.002). Extra-national vaccination plan alters microbiota composition itself, namely BCG, that influences *E. xylanophilum* (*p* = 0.003), flu vaccine, that interfere with *E. xylanophilum* concentration (*p* = 0.042) and anti-pneumococcal vaccine, that alters *S. salivarus* (*p* = 0.007), *D. longicatena* (*p* = 0.014) and *A. naeslaundii* (*p* = 0.002).

Moreover, Pearson’s correlations were applied with the aim to further analyze the possible interactions between demographic features and microbiota composition. The majority of correlations were validated regardless of the interaction with confounding factors. *Bifidobacterium adolescentis* seems to be significantly increased in women (*R* = 0.230; *p* = 0.021), while augmented weight decreases the concentrations of *A. naeslandii* (*R* = −0.429; *p* = 0.041) and *S. salivarus* (*R* = −0.420; *p* = 0.046). Antibiotic intake in the previous 6 months appears to decrease *Blautia luti* (*R* = −0.245; *p* = 0.039) and *R. litaburensis* (*R* = −0.268; *p* = 0.024), while probiotics/prebiotics/symbiotics rises the concentrations of *Clostridium_g26* spp. (*R* = 0.360; *p* = 0.001), *Enterobacterales* spp. (*R* = 0.286; *p* = 0.013), *Howardella* spp. (*R* = 0.391; *p* = 0.001), *E. coli* (*R* = 0.248; *p* = 0.032) and *Pantoea agglomerans* (*R* = 0.389; *p* = 0.032). As previously seen, vaccination has a huge impact on microbiota composition and so: BCG is positively correlated with *A. naeslandii* (*R* = 0.272; *p* = 0.018), but negatively correlated with *Fusicatenibacter* spp. (*R* = −0.238; p = 0.039) and *E. xylanophilum* (*R* = −0.257; *p* = 0.026). On the other hand, anti-pneumococcal vaccine is positively correlated with *A. naeslandii* (*R* = 0.354; *p* = 0.002) and *S. salivarus* (*R* = 0.307; *p* = 0.007), however inversely correlated with *Clostridium* spp. (*R* = −0.314; *p* = 0.005), *E. coli* (*R* = −0.260; *p* = 0.023) and *P. agglomerans* (*R* = −0.301; *p* = 0.008). Finally, the flu vaccine was correlated with increased levels of *A. naeslandii* (*R* = 0.277; *p* = 0.014), *Coprococcus comes* (*R* = 0.275; *p* = 0.014) and *F. prausnitzii* (*R* = 0.224; *p* = 0.047). Chronic diseases also modify the concentration of some bacteria. Arterial hypertension is correlated with increased levels of *A. naeslandii* (*R* = 0.252; *p* = 0.025) and *F. prausnitzii* (*R* = 0.238; *p* = 0.035), while diabetes mellitus type II rises the levels of *Ruminococcus* spp. (*R* = 0.354; *p* = 0.001), *R. trehalosifermentans* (*R* = 0.346; *p* = 0.002), *D. longicatena* (*R* = 0.276; *p* = 0.014) and *Sutterella wadswortemis* (*R* = 0.327; *p* = 0.003). Cancer also influences concentrations of *A. naeslandii* (*R* = 0.222; *p* = 0.049) and *R. litaburensis* (*R* = 0.270; *p* = 0.016). Chronic medication change microbiota composition as well, essentially anti-hypertensives, that increase levels of *Coprococcus* spp. (*R* = 0.294; *p* = 0.009), *A. naeslandii* (*R* = 0.227; *p* = 0.044) and *D. longicatena* (*R* = 0.229; *p* = 0.043), gastric protectors, that augments the concentration of *S. salivarus* (*R* = 0.253; *p* = 0.024) and, at last, antipsychotic drugs that influences *R. litaburensis* (*R* = 0.282; *p* = 0.012) and *R. timonensis* (*R* = 0.303; *p* = 0.007).

### Resistant enterotypes: What is the microbiota role?

Additionally, the susceptibility or resistance to acquire SARS-CoV-2 infection, influenced by intestinal microbiota composition was evaluated and it was observed the influence that some bacteria may have. *A. rectalis* (*p* = 0.017), *Gemminger formicilis* (*p* = 0.009), *E. coli* (*p* = 0.009), *P. agglomerans* (*p* = 0.034), *E. xylanophilum* (*p* = 0.020), *R. litaburensis* (*p* = 0.045) and *Roseburia cecicola* (*p* = 0.009) present a statistically significant difference between healthy and SARS-CoV-2 infected individuals. Moreover, *E. coli* (*R* = 0.224; *p* = 0.047) and *A. naeslandii* (*R* = 0.256; *p* = 0.023) seems to increase susceptibility to COVID-19, while *E. xylanophilum* (*R* = −0.278; *p* = 0.013) and *R. cecicola* (*R* = −0.260; *p* = 0.021) seems to protect against this infection.

Furthermore, in terms of increased infectivity, only *Dorea longicatena* (*p* = 0.020) had a statistically significant difference between participants with infected and uninfected house relatives.

### Microbiota signatures for the most common COVID-19 symptoms

Next, the correlation between microbiota composition and the most common COVID-19 symptoms was explored with the aim to predict, at long-term, the necessity to allocate ward and intensive care unit (ICU) beds for these patients. Therefore, as described in Table 2, several species had significant correlations.

**Table 2:** Correlation coefficients of microbiota signatures for the most common COVID-19 symptoms.

Symptom	Bacteria	Correlation coefficient	*p*-value
Dry cough	*A. rectalis*	0.232	0.039
	*G. formicilis*	0.228	0.043
	*R. trehasifermetans*	0.341	0.002
Dyspnea	*Coprococcus spp.*	0.232	0.012
	*P. xylanisolvens*	−0.259	0.021
	*A. naeslandii*	0.266	0.018
	*R. timonensis*	−0.265	0.018
	*R. cecicola*	−0.289	0.017
	*S. salivarus*	0.287	0.001
Fever	*Enterobacterales spp.*	0.280	0.012
	*E. xylanophilum*	−0.239	0.034
	*R. cecicola*	−0.269	0.016
Sputum	*B. xylanisolvens*	0.297	0.008
	*B. luti*	0.460	<0.001
Headache	*Howardella spp.*	0.302	0.007
	*G. formicilis*	0.277	0.013
	*E. coli*	0.256	0.023
	*R. trehasifermentans*	0.372	0.001
	*S. salivarus*	0.243	0.031
Myalgias and/or arthralgias	*Coprococcus spp.*	0.223	0.048
	*Streptococcus* spp.	0.234	0.038
	*A. naeslandii*	0.234	0.038
	*B. xylanisolvens*	0.276	0.014
	*D. longicatena*	0.237	0.039
Ageusia and/or anosmia	*Fusicatenibacter spp.*	0.247	0.028
	*Herbinix spp.*	0.233	0.039
	*Streptococcus spp.*	0.297	0.008
	*A. rectalis*	0.354	0.001
	*A. naeslandii*	0.297	0.008
	*G. formicilis*	0.377	0.001
	*B. xylanisolvens*	0.508	<0.001
	*B. adolescentis*	0.336	0.002
	*E. coli*	0.253	0.024
	*R. trehasifermentans*	0.522	<0.001
	*S. parasanguinis*	0.297	0.008
	*B. finegoldii*	0.401	<0.001
	*F. prausnitzii*	0.295	0.008
Odynophagia	*B. xylanisolvens*	0.237	0.035
	*C. comes*	0.246	0.029
Malaise	*Enterobacterales spp.*	0.304	0.006
	*Streptococcus spp.*	0.225	0.047
	*A. rectalis*	0.271	0.016
	*A. naeslandii*	0.225	0.047
	*G. formicilis*	0.297	0.008
	*B. xylanisolvens*	0.383	<0.001
	*E. coli*	0.348	0.002
	*R. trehasifermentans*	0.394	<0.001
	*S. parasanguinis*	0.225	0.047
	*P. agglomerans*	0.243	0.031
	*B. xylanophilum*	0.303	0.007
	*B. luti*	−0.239	0.003

Regarding the severity of dyspnea, it was demonstrated a statistically significant difference in *Coprococcus_g2* spp. (*p* = 0.047) and *R. cecicola* (*p* = 0.047), essentially between the need for mechanical ventilation and no ventilation at all. Also, *Enterobactelares* spp. (*R* = −0.325; *p* = 0.011) and *S. salivarus* (*R* = −0.325; *p* = 0.011) seems to decrease the necessity for mechanical ventilation.

## Discussion

Microbiota seems to be intrinsically correlated with immune system status. Therefore, the main aim of this study was to understand if microbiota can be used to predict the prognosis of COVID-19 and, thus, effectively allocate the proper quantity of medical resources to each patient.

First, there was the intention to evaluate the effect of microbiota on COVID-19 severity. So, we found a tendency to higher richness (Jackknife index) and diversity (Shannon index) in healthy patients, compared with severe and recovered groups, which presented a lower alpha-diversity, compatible with another study (Yamamoto et al., 2021). Also, it was observed that *Firmicutes/Bacteroidetes* (F/B) ratio was decreased in infected patients, especially in mild COVID-19, when compared with recovered patients and healthy volunteers. This inverse correlation between *Firmicutes/Bacteroidetes* ratio and COVID-19 severity was already observed in other related studies, as well as the higher difference in mild COVID-19 vs. severe COVID-19 (Moreira-Rosário et al., 2021; Yamamoto et al., 2021). The previously described low ratio is already established as a marker of microbiota dysbiosis, mainly seen in obese and Crohn’s disease patients. However, the fact that patients with milder symptoms have a lower F/B ratio than severe patients is really intriguing and might be explained by the fact that mild COVID-19 patients’ samples were collected in the acute period of the disease, while severe COVID-19 patients’ samples were collected after the acute period, when they were already in the ICU. Thus, these are two different periods in terms of microbiota resilience; where in the first period, microbiota is trying to respond to a pulse perturbation, and it is still testing its latitude. From this step, microbiota can cope with this stress and return to homeostasis, or it exceeds the stress threshold, namely its precariousness, and acquires a new state called dysbiosis (Alpuim Costa et al., 2021).

We discovered that almost 40% of severe COVID patients had bowel movement changes in the previous 6 months. We do not have any other information, however this might be used to support the proposed link between COVID-19 severity and the presence of gut dysbiosis prior to infection.

Furthermore, when comparing infected patients (mild and severe), healthy volunteers and recovered participants, we observed the following: (1) At *phylum* level, higher abundance of *Bacteroidetes* in the infected group; (2) At class level, higher quantity of *Bacteroidia* in infected patients and lower proportion of sequences of *Bacilli* in mild COVID-19 patients and healthy volunteers; (3) At the order level, higher amount of *Lactobacillales* in severe and recovered groups, as well as increased *Bacteroidales*, in the mild group; (4) At the family level, *Anaerovirgula*, *Streptococcaceae* and *Enterobacterales* were enriched in the severe group, whereas recovered group presented increased *Peptostreptococcaceae*; (5) At genus level, non-infected participants showed higher abundance of *Fusicatenibacter*, *Howardella* and *Ruminococcus*, while severe COVID-19 group had increased quantity of *Enterobacterales* and *Streptococcus* accompanied by lower levels of *Coprococcus_g2*, *Clostridium_g26*, *Herbinix*, *Ruminococcus* and *Ruminococcus_g2*. In terms of microbiota signatures, at the species level, we can observe an increased amount of *A. naeslundii* and *S. salivarus* in severe COVID-19 group, while mild patients had higher amounts of *B. xylanisolvens, E. coli, R. trehalosifermentans, P. agglomerans* and *B. finegoldii*. Recovered patients presented higher abundance of *B.luti, C. comes, D. longicatena, F. prausnitzii, R. litaburensis, R. timonensis* and *R. cecicola*, while healthy volunteers and increased levels of *A. rectalis, A. senegalensis, G. formicilis, B. adolescentis, P. xylanivorans, S. constellatus, S. parasanguinis, E. xylanophilum, D. longicatena, F. prausnitzii* and *S. wadsworthensis*. The above-mentioned bacterial species are *B. adolescentis*, a bacterium with the capacity to sustain the activation of nuclear factor-κB (NF-kB), *F. prausnitzii*, that is able to increase the expression of the anti-inflammatory cytokine IL-10, through induction of human colonic regulatory T cells, *E. rectale*, a bacterium that has been already associated with a diminution of inflammation in Alzheimer’s disease, *Ruminococcus obeum* and *D. formicigenerans*, also known by their anti-inflammatory properties (Zuo et al., 2020a). Moreover, *R. gnavus*, *R. torques*, *B. dorei* and *B. vulgatus* can be correlated to an increased dysregulation of the immune response since these bacteria already have a similar effect, described in inflammatory bowel disease (Villapol, 2020; Yeoh et al., 2021). Moreover, Zuo et al. (2020a) have demonstrated that the richness of some bacteria at the baseline can be correlated with disease severity. *Coprobacillus, Clostridium ramosum* and *C. hatheway* precede a more severe phenotype of COVID-19, alongside with lower concentrations of *F. prausnitzii* and *Alistipes onderdonkii* (Donati Zeppa et al., 2020; Villapol, 2020; Zuo et al., 2020a,b; Yamamoto et al., 2021). Likewise, other research projects demonstrated an enhanced quantity of *Proteobacteria*, accompanied by a reduction in short-chain fatty acids (SCFA)-producing bacteria from *Lachnospiraceae* family, essentially the genera *Roseburia* and *Lachonspira*. Also, there was a decreased plethora of the phylum *Actinobacteria*, specifically the genera *Bifidobacteria* and *Colinsella* (Moreira-Rosário et al., 2021). To highlight, *S. salivarus*, which is increased in severe COVID-19 patients, seems to reduce colonic α- diversity and decrease T helper (Th) cells related cytokines or transcription factors, namely IFN-γ (Th1), GATA-3 (Th2) and TGF-β (Treg) in mouse pups (Li et al., 2022). These characteristics can explain the promotion of dysbiosis and the increased susceptibility to cytokine storm. Furthermore, *B. xylanisolvens* and *E. coli*, present in the microbiota signature of mild COVID-19, have the ability to degrade k-carrageenan oligosaccharides, which induces pro-inflammatory effects in colon, therefore increasing dysbiosis, as well as COVID-19 prognosis (Yin et al., 2021). Additionally, Zhang et al. study further explored the functional profile of COVID-19 patients along the course of infection and reported that the pathways of SCFA and L-isoleucine biosynthesis were affected, as well as urea production was enriched. Therefore, these pathways modifications contributed to enhanced quantities of C- reactive protein, NT-pro-BNP and CXCL-10 (Zhang et al., 2022). Also, in terms of lipids metabolomics, other study detected a modification of lipids pathways between recovered and confirmed COVID-19 patients, where phosphatidylcholine, phosphatidylethanolamine and diglyceride were enhanced, while sphingomyelin and monoglyceride were diminished (Ren et al., 2021).

Moreover, we explored the demographical characteristics of the population on the influence of COVID-19 prognosis, as well as in the microbiota composition itself. Regarding the main demographic features, we found out that the impact of COVID-19 severity is summed up in the following statements: (1) ethnicity; (2) chronic diseases; (3) antibiotic intake; (4) vaccination and are in line with the literature. Firstly, ethnicity is already described as an important factor of prognosis in a wide range of diseases, especially African ethnicity. It appears that this ethnicity has reduced vitamin D activation, which is associated with a worse COVID-19 prognosis, although it cannot be dissected if it is due to genetic conditions or socioeconomical factors (Rhodes et al., 2021). Then, some established risk factors for more severe COVID-19, namely hypertension, diabetes mellitus type II and obesity (Moreira-Rosário et al., 2021), were already replicated in our results. Moreover, antibiotic intake was associated with a higher risk of severe COVID-19, which was also described by Llor et al. (2021). Finally, we came across a significant impact of vaccination, which includes the normal Portuguese National Vaccination Plan, BCG, anti-pneumococcal and common flu, in the outcome of COVID-19. It has been shown in Gonzalez-Perez et al. study, that BCG and other vaccines may influence the severity of COVID-19, by inducing the potentiation of innate immune system response and, therefore, increasing the defense capacities against microorganisms, namely SARS-CoV-2 (Gonzalez-Perez et al., 2021).

Since microbiota dysbiosis might influence COVID-19 severity, can it explain why some people are more resistant to infection? Also, why can some people spread the virus more effectively than others? One study might have the answer to both questions (Zuo et al., 2021). The study reports that during the hospital stay, the gut microbiota of patients was enriched with *Bacteroides dorei*, *B. thetaiotaomicron*, *B. massiliensis* and *B. ovatus*, which are known for downregulation of ACE2 receptors in murine model. This downregulation increases SARS-CoV-2 replication efficiency and so, the enhanced concentration of the above-mentioned bacteria is correlated with an augmented fecal SARS-CoV-2 load. Contrarily, *Firmicutes Erysipelotrichaceae bacterium 2_2_44A*, upregulates ACE2 receptor, thus originating a decreased fecal viral load. For instance, the described modifications were tested *in vitro* and, consequently, a high infectivity SARS-CoV-2 signature arose, as well as a low-to-no infectivity SARS-CoV-2 signature. The first one is composed by *Collinsella aerofaciens*, *C. tanakaei*, *S. infantis* and *Morganella morganii*, a group of opportunistic pathogens that grow after microbiota dysbiosis. The second signature is composed by *Parabacteroides merdae*, *B. stercoris*, *A. onderdonkii* and *Lachnospiraceae bacterium*, which are SCFA- producing bacteria, that decreases the chance of microbiota dysbiosis, as well as hold the capacity to compete with opportunistic pathogens (Lee et al., 2020; Alpuim Costa et al., 2021; Moreira-Rosário et al., 2021). Furthermore, Ke et al. (2022) study, might shed a light on how gut microbiome might facilitate the entrance of SARS-CoV-2 into the cells. It was reported that one important pathway, the pentose phosphate, was enhanced in COVID-19 patients, when compared to healthy volunteers, simultaneously with the bacteria *Hungatella effluvia* and *Enterocloster bolteae*, which might contribute to this increased pathway usage. The pentose phosphate pathway is important in cell-to-cell and receptor-dependent virus-cell fusion, which indicates that some bacteria might contribute to increase SARS-CoV-2 attachment and, subsequently, entrance in cells, increasing SARS-CoV-2 infectivity (Ke et al., 2022). Our study added a phew more bacteria that may increase infectivity, since it is significantly more present in infected COVID-19 patients, namely *E. coli*, *A. naeslandii* and *D. longicatena*. On the other hand, there were some other SCFA-producing bacteria than can augment the low-to-no infectivity microbiota signature, such as *A. rectalis, G. formicilis, P. agglomerans, E. xylanophilum, R. litaburensis* and *R. cecicola*. To highlight that *D. longicatena* can influence the whole microbiota ecosystem in intestines by enhancing gut mucosa inflammation (Nishida et al., 2017), which *per se* increases ACE2 receptor expression, facilitating SARS-Cov-2 infection and virus proliferation (Li et al., 2021).

At last, we found out that the most common COVID-19 symptoms are correlated with specific microbiota signatures (Table 2) that may be used to predict patient’s clinical outcomes. It is important to emphasize that anosmia is positively associated mostly with SCFA-producing bacteria. One study revealed that SCFA can change olfactory perception and reduce the sense of smell, therefore, leading to hyposmia and anosmia. Since scent is intrinsically correlated with flavor perception, it can also induce dysgeusia (Koskinen et al., 2018). Although the literature does not hold any other explanation of these symptomatic microbiota signatures, their association might be enlightened, by the influence of the communication in an axis between the affected organ and intestinal microbiota. On the other hand, unspecific symptomatology (e.g., malaise) could be a result of the interaction between microbiota metabolites and the immune system.

This project has demonstrated that microbiota has an impact on human’s health and disease. That is why, hereby, we propose a pipeline, with the aim to increase efficiency in a pandemic, in disease triage and better allocate our finite resources to avoid hospitals overwhelming at the cost of losing lives. First, there is the need to do a population fecal material collection of people accompanied by a demographic questionnaire, with the aim of creating a microbiota database. Then, when a newly emergent disease arises, a fecal material collection of patients with different degrees of severity is assembled to understand the impact of this new disease on the microbiota and the opposite by comparing the microbiota of infected patients with the healthy microbiota of the same patient, present in the microbiota database. Then, NGS sequencing is performed and the differences between each microbiota are obtained. Furthermore, there is the possibility of identifying bacterial biomarkers that can impact positively or negatively the prognostic of the patient. With this information, it is possible to triage the patients more efficiently at the moment of diagnosis and allocate the necessary medical resources by developing a quick test. Also, there is the possibility of manipulating microbiota through probiotics, prebiotics, symbiotics or even fecal transplant of microbiota, with the aim of modifying the prognosis and decreasing disease severity.

Soon, it is important not only to explore the bacterial community within microbiota, but also the fungus and viruses and how they interact with SARS-CoV-2. Moreover, it is crucial to understand the metabolomics of this complex interplay between the organism, gut microbiota and SARS-CoV-2, especially tryptophan, which is the subtract for gut microbiota and appears to be an indirect biomarker of intestinal epithelium RAS-independent ACE2 receptors activity.

Furthermore, there is a need to explore the relationship between SARS-CoV-2 and EBV. That is due to the fact that some studies found that there is a high incidence of EBV/SARS-CoV-2 co-infection or reactivation of latent EBV infection, which seems to be correlated with worse COVID-19 prognosis (Meng et al., 2022).

At last, since the intrinsic relation between gut microbiota and immunity system is a well-known assumption, it is necessary to correlate the genetic polymorphisms of our immunity system, which had already been associated with poorer COVID-19 prognosis, with gut microbiota modifications, in order to correctly stratify patients in terms of risk prognosis.

## Conclusion

This study highlights the importance of microbiota in the triage and prognosis definition in COVID-19 patients, allowing a more efficient allocation of medical resources. However, it has some limitations, mainly because the control group is small and not age-matched. Also, this study was developed during the first two waves of COVID-19 in Portugal and so, since the SARS-CoV-2 variants had changed and vaccination was already performed, there is a need to be more careful when extrapolating our results. Other limitations were related to the fact that we had a total of 104 fecal samples, while only 87 demographic questionnaires were fulfilled, which restricts the control of confounding variables. Also, it would be helpful to correlate our data with serum and fecal SARS-CoV-2 viral load, as well as with laboratory data, such as CPR, Procalcitonin, LDH, leukocyte differentiation and D-dimers.

This type of investigation gives the ability to predict whether a patient will have milder/more severe symptoms or long-term consequences from the COVID-19 infection. Soon, it will be possible to use fecal microbiota transplantation in order to change the outcome of the patient having more severe symptomatology or even change this outcome at the moment of diagnosis.

## Data availability statement

The data presented in this study are deposited in the Sequence Read Archive of NIH, accession number PRJNA887817 (https://www.ncbi.nlm.nih.gov/sra/PRJNA887817).

## Ethics statement

The studies involving human participants were reviewed and approved by the study was reviewed and approved by the Ethical Committee of ESTeSL (CE-ESTeSL-Nº.28-2020), Ethical Committee of Hospital de Cascais (EO 14/2020), Ethical Committee of Hospital Garcia da Horta (ref 18/06/2020) and Ethical Committee of Hospital da Luz (ref. 16/07/2020). The patients/participants provided their written informed consent to participate in this study.

## Author contributions

JN, MD, MB, and DAC: conceived the study and oversaw the sample acquisition and maintenance. JN, MD, MB, JM, ML, AP, FT, MR, and CS: contributed to data collection and analysis. JN and MD participated in manuscript writing. JN and DAC: oversaw clinical sample. MD, MB, and CS: oversaw the data collection. All authors contributed to the article and approved the submitted version.

## Funding

The authors acknowledge financial support from Instituto Politécnico de Lisboa that supported this project with the grant Microcovid. This project was also partially supported by FCT/MCTES (UIDB/05608/2020 and UIDP/05608/2020).

## Conflict of interest

The authors declare that the research was conducted in the absence of any commercial or financial relationships that could be construed as a potential conflict of interest.

## Publisher’s note

All claims expressed in this article are solely those of the authors and do not necessarily represent those of their affiliated organizations, or those of the publisher, the editors and the reviewers. Any product that may be evaluated in this article, or claim that may be made by its manufacturer, is not guaranteed or endorsed by the publisher.
